# Current State and Future Directions of Technology-Based Ecological Momentary Assessment and Intervention for Major Depressive Disorder: A Systematic Review

**DOI:** 10.3390/jcm8040465

**Published:** 2019-04-05

**Authors:** Desirée Colombo, Javier Fernández-Álvarez, Andrea Patané, Michelle Semonella, Marta Kwiatkowska, Azucena García-Palacios, Pietro Cipresso, Giuseppe Riva, Cristina Botella

**Affiliations:** 1 Department of Basic Psychology, Clinic and Psychobiology, Universitat Jaume I, Av. Sos Baynat, s/n, 12071 Castellón, Spain; azucena@uji.es (A.G.-P.); botella@uji.es (C.B.); 2 Department of Psychology, Università Cattolica del Sacro Cuore, Largo Gemelli, 1, 20100 Milan, Italy; javier.fernandezkirszman@unicatt.it (J.F.-Á.); pietro.cipresso@unicatt.it (P.C.); giuseppe.riva@unicatt.it (G.R.); 3 Department of Computer Science, University of Oxford, Wolfson Building, Parks Rd, Oxford OX1 3QD, UK; andrea.patane@cs.ox.ac.uk (A.P.); marta.kwiatkowska@cs.ox.ac.uk (M.K.); 4 Applied Technology for Neuro-Psychology Lab, IRCCS Istituto Auxologico Italiano, 20149 Milan, Italy; semonellamichelle@gmail.com; 5 CIBER Fisiopatología Obesidad y Nutrición (CIBERobn), Instituto Salud Carlos III, 28029 Madrid, Spain

**Keywords:** major depressive disorder, ecological momentary assessment, ecological momentary intervention

## Abstract

Ecological momentary assessment (EMA) and ecological momentary intervention (EMI) are alternative approaches to retrospective self-reports and face-to-face treatments, and they make it possible to repeatedly assess patients in naturalistic settings and extend psychological support into real life. The increase in smartphone applications and the availability of low-cost wearable biosensors have further improved the potential of EMA and EMI, which, however, have not yet been applied in clinical practice. Here, we conducted a systematic review, using the Preferred Reporting Items for Systematic Reviews and Meta-Analyses (PRISMA) guidelines, to explore the state of the art of technology-based EMA and EMI for major depressive disorder (MDD). A total of 33 articles were included (EMA = 26; EMI = 7). First, we provide a detailed analysis of the included studies from technical (sampling methods, duration, prompts), clinical (fields of application, adherence rates, dropouts, intervention effectiveness), and technological (adopted devices) perspectives. Then, we identify the advantages of using information and communications technologies (ICTs) to extend the potential of these approaches to the understanding, assessment, and intervention in depression. Furthermore, we point out the relevant issues that still need to be addressed within this field, and we discuss how EMA and EMI could benefit from the use of sensors and biosensors, along with recent advances in machine learning for affective modelling.

## 1. Introduction

Major depressive disorder (MDD) is a common debilitating psychiatric disease characterized by mood disturbances, loss of interest and pleasure in daily activities, disturbed appetite and sleep, loss of energy, and psychomotor retardation or agitation. According to the World Health Organization, depression is one of the leading causes of disease and disability in the world, annually affecting 4.4% of the general adult population [1]. In addition to producing high costs for the public health system, depression seriously impairs patients’ functioning, leading to increased mortality, high suicide rates, exacerbated medical conditions, and high consumption of alcohol and illegal drugs [2,3,4,5].

As a result of the increased availability of smartphones and portable and wearable devices, a growing body of research has begun to explore new digital technologies as potential tools to foster assessments and interventions in clinical practice. More specifically, technology-based ecological momentary assessment (EMA) and ecological momentary intervention (EMI) have been proposed as alternative strategies to assess patients ecologically in naturalistic settings and deliver psychological support in daily life.

### 1.1. Ecological Momentary Assessment

Traditional clinical assessments are based on retrospective self-reports in which patients are asked to summarize their symptoms and affective experiences over the past few weeks. Nevertheless, increasing evidence shows that these tools are not able to capture MDD dynamics, such as symptom fluctuations or mood shifts over time [6,7]. Likewise, self-reports are affected by recall bias. In other words, depressed patients have been found to alter the content of past experiences when asked to retrieve them retrospectively [8,9], judging symptoms as more severe [10] or increasing the elaboration of negative information [11]. 

EMA emerged as an alternative assessment strategy to better grasp affective and behavioural dynamics in daily life [12,13,14]. Not surprisingly, a growing body of research has applied this approach to exploring mood disorders [15,16]. On the one hand, the term “ecological” refers to the environment where the data are collected. Behaviours, thoughts, and affect are repeatedly written down in real-world contexts. On the other hand, the term “momentary” refers to the focus of the assessment, i.e., close in time to the experience. The first studies to use this approach adopted paper-and-pencil daily diaries, but the discomfort, low compliance, and low experimental control over backfilling made them not very efficacious [14]. The exponential progress of information and communication technologies (ICTs) and the increasing availability of smartphones offered novel opportunities to ecologically assess patients. On the one hand, mobile technologies allow the shortcomings of traditional diaries to be overcome by eliminating the need for manual data entry and by increasing control on backfilling, thus obtaining more accurate data. On the other hand, all the necessary processes can be integrated in one tool, for instance, a smartphone, thus decreasing intrusiveness and increasing users’ comfort, and providing a more engaging and dynamic experience. During the day, indeed, patients are automatically prompted by the device to fill in self-reports that are subsequently stored and safely sent to clinicians and/or researchers. More recently, the potential of EMA was extended due to the integration of self-reports with data gathered from embedded sensors and wearable biosensors, hence allowing for a multimodal approach. Unobtrusive wearable biosensors can continuously monitor physiological parameters throughout the day with high precision [17], whereas smartphone embedded sensors make it possible to indirectly collect data about patients’ behaviours and habits, such as their social media use, physical activity, or social interactions [18,19]. Overall, the integration of these tools has the potential to revolutionize traditional assessments, leading to the exploration of new facets of MDD obtained in daily life contexts that are often difficult to capture in laboratory settings. 

### 1.2. Ecological Momentary Intervention

According to statistics, 70% of people suffering from mental disorders do not receive adequate psychological treatment or reach complete clinical remission [20]. Affordances of technological developments, as Kazdin and Blase suggested, may facilitate new solutions for disseminating evidence-based psychotherapy [21].

The same “ecological” and “momentary” principles have been applied to the development of innovative interventions (EMI) [22] that go beyond traditional clinical settings and extend the delivery of psychological support into real life [23]. EMI has the advantage of providing psychological support directly on hand-held mobile technologies during the flow of daily experiences, in real-time settings, and at specific time points in the day, without the need for face-to-face meetings with a clinician [24]. EMIs can be delivered both as stand-alone treatments or in combination with other treatments. Moreover, similarly to EMAs, the use of data gathered from biosensors and embedded sensors along with machine learning techniques can increase the customization of the proposed interventions [16,25].

### 1.3. Objectives

Recent studies have confirmed the feasibility of mobile health (mHealth) applications and patients’ interest in and adherence to these technologies, suggesting the great potential of this approach in the clinical field [23,26]. Nevertheless, no systematic review has explored technology-based EMA and EMI for MDD to date. Although two reviews focused on EMAs for mood disorders [15,16], most of the included studies were based on paper-and-pencil daily diaries, and the target population included adults and adolescents with bipolar disorder (BD) and borderline personality disorder (BPD). 

Coinciding with our field of interest, the aim of this systematic review is to provide an overview of the state of the art of technology-based EMA and EMI for MDD from both a clinical and technological point of view. Our final objective is to show how and why clinical practice could benefit from the use of these approaches. In doing so, we will describe the potential of new technologies in this field, and we will discuss how EMAs and EMIs could be performed with sensors and biosensors along with recent advances in machine learning for affective modelling.

## 2. Methods

Preferred Reporting Items for Systematic Reviews and Meta-Analyses (PRISMA) criteria [27] were followed. For the systematic review protocol, see [28].

### 2.1. Search Strategy

To collect relevant publications, a computer-based search was performed (March 2019). We searched in two high-order databases, PubMed and Web of Science (Web of Knowledge), using the following string: ((EMA) OR (“ecological momentary assessment”) OR (EMI) OR (“mobile health”) OR (mhealth) OR (smartphone) OR (“ecological momentary intervention”) OR (ESM) OR (“experience sampling method”) OR (“ambulatory assessment”) OR (“personal digital assistant”) OR (“ambulatory monitoring”) OR (“real time data capture”) OR (“real time monitoring”) OR (“real time interventions”) OR (“electronic diary”) OR (“repeated observations”) OR (“diary data”) OR (“time series”)) AND ((“affective disorder”) OR (“mood disorder”) OR (depress*) OR (depression) OR (MDD) OR (“major depressive disorder”) OR (“major depression”) OR (“unipolar depression”) OR (“affective symptoms”).

This search produced a total of 4993 articles. After eliminating duplicate papers, we made a first selection by reading titles and abstracts, and 401 articles were retrieved. We finally selected publications by applying the selection criteria described in the following paragraph, obtaining 40 papers. 

**Figure 1:**
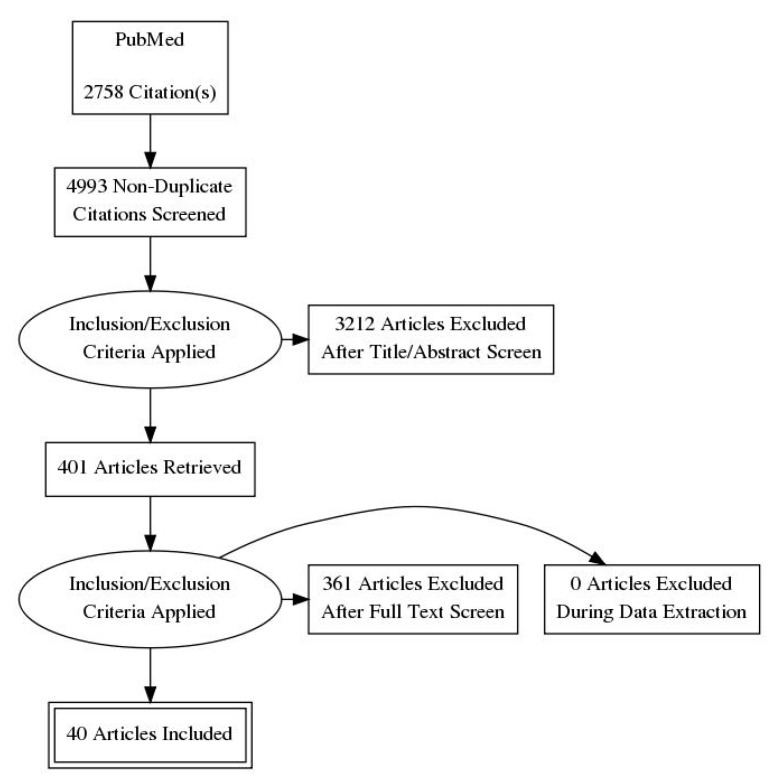
PRISMA (Preferred Reporting Items for Systematic Reviews and Meta-Analyses) flow diagram.

Three individual researchers (D.C., J.F.-Á., and M.S.) performed the search for publications in the English language. More details are provided in Table 1 and in the flow diagram (Figure 1), in order to make this search replicable in the future.

### 2.2. Selection Criteria

We included all studies involving a sample of adults with a primary (both current or past) diagnosis of MDD, using recognised diagnostic criteria (Diagnostic and Statistical Manual of Mental Disorders—DSM; International Classification of Disease—ICD). We excluded non-English papers and studies that did not meet the inclusion criteria. We also excluded articles that did not have full-text available, and the following types of manuscripts: Conference papers, reviews and systematic reviews, metanalyses, meeting abstracts, notes, case reports, letters to the editor, editor’s notes, extended abstracts, proceedings, patents, editorials, and other editorial materials. We tried to contact the corresponding authors, when necessary, to obtain missing or supplementary data.

Ecological momentary assessment: We included studies that adopted an ecological momentary assessment by means of hand-held technologies (such as smartphones, personal digital assistants, or hand-held computers) for the collection of daily self-reports, thus excluding studies that used paper-and-pencil diaries. Additionally, we included studies that integrated daily self-reports with data supplied by sensors and biosensors.

Ecological momentary interventions: We included EMIs that were provided to patients through hand-held technologies. We selected studies in which the proposed EMI was either a stand-alone intervention or combined with other types of treatment. We also included EMI that collected data from wearable biosensors or device-embedded sensors. Because providing continuous feedback to patients has been shown to be a valuable therapeutic procedure [29], we also included studies that adopted EMA-based feedback as a therapeutic tool for clinically depressed patients.

### 2.3. Quality Assessment and Data Abstraction 

To control for the risk of bias, PRISMA recommendations for systematic literature analysis were followed. Studies were independently selected by three different authors (D.C., M.S., and J.F.-Á.), who first analysed titles and abstracts and subsequently selected the full papers that met the inclusion criteria, resolving disagreements through consensus. For what concerns the EMA included studies, the main aim was to provide a perspective of clinical, technical, and technological issues related to this approach: In other words, we were interested in EMA as a clinical and experimental tool to be used in the psychological field, regardless of the study design or variables of outcome (Colombo et al., 2018). No risk of bias assessment was therefore performed. Differently, risk of bias of EMI studies was assessed by two independent reviewers (D.C and J.F.-Á.). As both randomized and non-randomized controlled trials were included, quality assessment was assessed with the Downs and Black quality index [30]. 

The data extracted from each study were as follows: Author(s), sample(s), variable(s), device(s), sensor(s), duration, prompt(s) per day, sampling schema, primary outcome(s) for the selected studies on EMA (Table 2); and author(s), name of the intervention, sample(s), content of the intervention, duration, device(s), sensor(s), and primary outcome(s) for the studies proposing an EMI (Table 3).

## 3. Results

### 3.1. Ecological Momentary Assessment in MDD

After applying the inclusion criteria, 32 studies were retrieved that investigated and assessed MDD through a technology-based EMA. 

A synthesis of the results is provided in Table 2.

#### 3.1.1. Electronic Devices and Use of Sensors

Most of the selected studies administered daily self-reports either through a personal digital assistant (PDA) or a smartphone. Only three studies adopted different technological solutions that allowed them to collect both self-reports and data gathered from sensors and biosensors. Conrad et al. [31] used the LifeShirt System (Vivometrics, Inc., Ventura, CA, USA), a comfortable garment with integrated biosensors that can continuously monitor various cardiopulmonary parameters, including heart rate (HR), respiration, and posture. With an embedded hand-held computer, patients can also complete self-reports following daily beep signals. In another study, Kim and colleagues adopted ECOLOG [32], a watch-type computer characterized by an 8-direction joystick and an integrated actimetry sensor. Via a beep signal, the wristwatch prompts patients to complete momentary assessments directly on the watch screen. Similarly, a compact wrist–worn electronic diary was used by Littlewood et al. to collect both self-reports and sleep/wake cycles with an embedded actimetry sensor [33]. 

Although a growing number of studies analyse data from embedded-sensors and biosensors in research on mental health disorders [34], their use in association with EMA has been low in the field of MDD. Among our selected studies, only seven of the 32 studies collected physiological measures in addition to self-reports. Conrad and colleagues collected cardiac and respiratory measures as indices of vagal activity, along with physical activity measured through an embedded actimetry sensor [31], whereas Ottaviani and colleagues collected ambulatory HR [35]. The remaining five articles investigated the association of depressive symptoms with sleep/wake cycles [32,33,36] and physical activity [37,38] using actimetry sensors. 

#### 3.1.2. Sampling Methods

Currently, different EMA designs can be used to define prompt scheduling, depending on the main purpose of the study. It is possible to prompt participants using fixed time periods or randomized/semi-randomized samplings (time-based sampling). Alternatively, participants can be asked to personally fill in the assessment after the occurrence of a specific behaviour or event (event-based sampling). Whereas time-based samplings depend on a signal emitted by the device (signal-contingent), event-based samplings are not preceded by a prompt (event-contingent). Signal-contingent schemas are useful when repeated measures are needed to obtain a representative value of a variable or when the objective is to capture dynamic variables (e.g., mood), whereas event-contingent schemas are more likely to be adopted when the main focus is on a specific behaviour that occurs randomly or less frequently during the day (e.g., smoking a cigarette). Regarding our selected studies, none of them adopted event-based sampling. Most of the studies collected data using randomized or semi-randomized schemas, whereas nine studies prompted participants to note information at fixed time points during the day. This latter approach was adopted especially by the studies that investigated the association between cortisol or melatonin and depression, i.e., when the assessed variable required greater temporal precision and accuracy. 

The duration of the data collection showed great variability. Some studies collected self-reports for a brief time period (less than 3 days); this choice was especially observed in the field of cortisol and sleep pattern research. Other studies required longer periods of assessment, where participants were involved for one or two months. This was especially true for studies investigating physical activity and its association with depressive symptoms. The same high variability was observed in the number of prompts, which varied from 1 to 20 prompts per day.

#### 3.1.3. Compliance and Dropout Rates

With the term “compliance”, we refer to the percentage of answered prompts. A few studies did not report this information [35,40,41,42,51,57,60]. However, the majority clearly addressed this issue. Sixteen studies reported compliance rates higher than 85%, five studies showed rates between 84% and 70%, and four studies collected 65% of the total possible answers. Patient dropout was related to diagnosis change, subjective burden, technical problems, incomplete data, retrospective completion of the electronic diary, missed prompts, worsening of symptoms, or non-attendance at follow-up sessions. 

To prevent backfilling, different solutions were adopted. In most of the studies, participants could complete self-reports for a fixed time period after the prompt, ranging from a few minutes to a maximum of one hour. To increase compliance, two studies also gave participants the possibility of postponing prompts.

#### 3.1.4. Contribution of EMA to the Study of MDD

As Table 4 shows, so far EMA has been applied to seven different fields. In the following paragraph, we will provide an overview of EMA’s contribution to the understanding and assessment of MDD. 

##### Recall Bias

Increasing evidence shows that memories often have inaccurate and imprecise content due to recall bias. In the case of EMAs, two studies were carried out to investigate this bias, comparing EMA daily data to retrospective assessments. Ben-Zeev and colleagues compared positive (PA) and negative (NA) affect collected through an EMA to scores obtained by means of traditional paper-and-pencil retrospective questionnaires [8]. When retrospectively recalled, both PA and NA were overestimated, regardless of the diagnosis. Interestingly, the control group was more likely to exaggerate the retrieval of PA rather than NA, but this trend was not observed in depressed patients. By contrast, Torous and colleagues developed a smartphone application to administer randomized subsets of items taken from the Patient Health Questionnaire (PHQ-9) [69], compared to the traditional paper-based PHQ-9. Symptoms were evaluated as more severe in daily EMA evaluations, compared to the retrospective PHQ-9 assessment. According to the authors, this discrepancy could be due to different factors, such as recall bias or stigma. 

##### Symptom Monitoring

Unexpectedly, we could only retrieve three studies within this research field, i.e., studies that actually applied EMA to monitor clinically depressed patients. Husky and colleagues investigated the acceptability of a three-days computerized ambulatory monitoring on MDD and BD patients, showing encouraging compliance and acceptance rates among both samples. Practice effects were observed (faster response time over the course of the study), thus suggesting the importance of considering the potential effects of EMA duration on self-reports [39]. Schaffer et al. developed a system called “Mental Health Telemetry” to monitor symptoms of patients receiving pharmacological treatment [40]. According to the results, a reduction in depressive symptoms was already observable one day after beginning the treatment, and symptoms on day 7 were predictive of treatment outcome. Similarly, iHOPE is a smartphone application for the daily monitoring of depressive symptoms and sleep patterns [41]. EMA assessments of depression, sleep quality, and anxiety were highly associated with the Hamilton Depression Rating Scale (HAM-D), administered at baseline. Nevertheless, application use decreased significantly over the weeks, from 3.4 days per week to 0.4 days per week after 8 weeks, highlighting the important issue of compliance in EMA assessments. 

##### Cortisol Secretion

Stetler and colleagues investigated the associations among cortisol and sleep patterns, social interactions [43], and daily activities [42]. Not only were cortisol levels after awakening different in depressed and healthy participants, but the impact of psychosocial variables on cortisol secretion was also dissimilar. Consistently, the Hypothalamic–pituitary–adrenal (HPA) axis of depressed patients was no longer able to respond to the timing of the sleep-wake cycle, daily routines, and external social experiences. One study explored the impact of cortisol on affect, showing a bidirectional association between PA and NA and daily cortisol levels [45]. Nevertheless, high variability was observed among participants regarding the timing, direction, and sign of this association. For instance, NA was positively associated with cortisol 50% of the time, while the association between cortisol and PA was almost always negative. Booij et al. identified higher cortisol and α-amylase levels among depressed individuals [36]. Similarly, when applying individual correction for lifestyle factors, the association of depression to cortisol and the ratio of α-amylase over cortisol was no longer significant, suggesting that generalization from groups does not always reflect the single individual. Nevertheless, Conrad and colleagues could not find cortisol differences between depressed and non-depressed participants. Interestingly, a negative correlation between NA and heart rate variability (HRV) was observed only in the control group, suggesting that constant NA may alter the normal interaction between affectivity and the autonomic nervous system [31]. 

Finally, interesting outcomes were also observed among remitted MDD patients [44]. Despite remission, patients showed reduced cortisol levels throughout the day and a different interaction between affect and cortisol, thus suggesting a reduction in the HPA axis’ responsiveness as a potential marker of recurrent depression.

##### Sleep Patterns

According to our search, six studies adopted an EMA to explore sleep disturbances in depression. Through the daily administration of morning self-reports about sleep patterns, O’Leary et al. found that depression was associated with lower perceived sleep quality, which in turn affected negative emotional reactivity to both neutral and unpleasant events during the day [47]. However, in healthy participants, sleep disturbances only affected emotional reactivity to unpleasant events. In other words, depression could be a factor affecting the relationship between sleep quality and emotional reactivity. Similarly, two studies analysed the influence of sleep quality on daily affect [46,48]. As expected, higher sleep quality was associated with higher PA in both healthy and depressed participants. Surprisingly, there was no evidence of the moderating role of depression in the association between sleep and affect. Nevertheless, sleep quality affected daily mood, but not vice versa, because higher sleep quality was associated with increased PA and decreased NA the following day. This association did not differ between depressed and healthy participants. Similarly, sleep duration was found to affect next-day physical activity, but again, no difference between depressed and non-depressed individuals was observed [38]. An EMA was finally adopted to investigate the association between sleep patterns and suicide ideation in a sample of depressed patients [33]. Poor sleep quality, both at subjective and objective levels, was associated with increased suicide ideations the following day. However, suicidal thoughts did not predict sleep patterns the following night.

Bouwmans and colleagues also collected repeated saliva samples to analyse the association of depression with melatonin, an important hormone related to sleep onset [49]. A bidirectional relationship between affect and fatigue, and melatonin was pointed out: Melatonin is associated with changes in affect and fatigue; however, affect and fatigue are also predictors of melatonin levels. Participants that did not show this association were likely to report higher rates of depression, worse sleep quality, and lower energy expenditure. 

##### Physical Activity

In order to analyse the effect of self-initiated physical activity on mood, clinically depressed patients were asked to report their daily physical activity [50]. Both healthy and depressed participants showed higher levels of PA following physical activity, but no decrease in NA. Notably, the increase in PA after physical exercise was greater in depressed patients, which is consistent with the ample evidence supporting behavioural activation in general, and physical activity in particular, for the treatment of depression. Confirming these results, another study found that physical activity was associated with subsequent increased PA, regardless of the diagnosis [37]. However, the analysis also revealed high subjective variability in the association between physical activity and mood in terms of strength, direction, and temporal aspects. Finally, Kim and colleagues developed a statistical model with cross validity that identified a significant association between higher intermittency of locomotor activity and worse mood ratings [32], suggesting the possibility of predicting patients’ moods through the analysis of momentary locomotor patterns. According to their model, a worsening of depressive mood was associated with increased intermittency of locomotor activity. 

##### Rumination

Ruscio and colleagues investigated the relationship between stressful events and rumination in MDD and GAD patients [52]. Both clinical samples showed higher levels of rumination in response to stressful situations, which were further worsened by symptom severity and extensive comorbidity. In addition, rumination significantly mediated the impact of stress on symptoms and affect; that is, higher rumination after a stressful event predicted greater NA and more maladaptive behaviours. Putman and colleagues investigated rumination and self-esteem through the assessment of resting baseline PFC alpha activity, along with the momentary assessment of affect and depressive symptoms, in a sample of clinically depressed individuals [51]. Rumination was found to be associated with an increased alpha signal in the bilateral prefrontal cortex (i.e., decreased neural activation), whereas an increased alpha signal in the right prefrontal cortex was positively correlated with higher self-esteem ratings. One study investigated perseverative thoughts (i.e., depressive rumination, worry, and reactive rumination) in relation to mind wandering [35]. Participants were instructed to complete a smartphone diary every 30 min for one day, and these self-reports were integrated with continuous HR monitoring. Confirming the hypothesis that mind wandering is not a maladaptive behaviour per se, only perseverative cognition was associated with health risk factors, such as lower HRV, worse mood, and higher interference in daily functioning. Finally, one study examined the dynamics of worry and rumination in daily life [53]. Contrary to the hypothesis, levels of worry were not significantly associated with the occurrence of significant events, whereas rumination was significantly higher in response to these circumstances. Compared to the control group, clinically depressed individuals showed decreased PA and increased NA as a consequence of high rumination levels.

##### Affect and Emotional Reactivity

Thompson and colleagues investigated emotional reactivity, emotional inertia, and emotional instability in depressed patients [50]. Compared to healthy participants, clinically depressed patients showed higher NA instability, whereas no differences in PA instability were observed. Both samples reported increased NA after a negative event; however, depressed patients showed a greater decrease in NA and increase in PA after a positive event. These results were confirmed by another study that showed a greater reduction in NA following positive events in depressed individuals [55]. When considering BPD comorbidity, depressed patients were found to be less emotionally influenced by events, and to perceive themselves as less emotionally reactive [56]. Other factors that affect emotional reactivity are gender and past depression [54]. In one study, women and remitted patients evaluated daily events as more negative than men, and they showed worse mood and higher emotional reactivity in response to daily stressors. Finally, a smartphone application was developed to assess visual mental imagery and its impact on mood and affective reactivity in healthy people and remitted MDD patients [57]. Participants were asked to focus on their mental representations, i.e., what they had in mind, eight times per day. Imagery-based processing was associated with better mood, regardless of the valence of the mental representation. This pattern was similar in healthy and depressed participants. However, no association between mental imagery and affective reactivity was observed. 

Regarding daily affect, one study explored the impact of gambling desire on mood in a sample of depressed individuals [59]. Higher levels of sadness and arousal were associated with higher rates of gambling desire. Consistently, depressed participants were also likely to perform gambling behaviours to increase their current PA levels. However, momentary affect did not predict actual gambling behaviours. An EMA was also used to investigate the influence of social rejection and disagreement on daily affect in MDD and BPD patients [58]. As expected, momentary and daily negative interpersonal events triggered higher NA (fear, hostility, and sadness) in both groups. High levels of hostility predicted rejection and disagreements, whereas sadness was only a predictor of social rejection. The aforementioned relationships were stronger in BPD patients than in depressed participants.

Finally, one study investigated the topology and temporal dynamics of depression and anxiety symptoms using contemporaneous and temporal network models [60]. Positive (positive, content, enthusiastic, energetic) and negative (down) mood were the most representative variables of patients’ core symptoms. While “worried” and “down” did not show temporal influence, “positive mood”, “hopelessness”, “anger”, and “irritability” were the strongest drivers of moment-to-moment symptomatology.

### 3.2. Ecological Momentary Intervention in MDD

The selection process resulted in eight studies that administered an EMI to clinically depressed patients. In all, four different interventions were identified: Psymate, Mobylize, Hel4Mood, and Medlink.

#### 3.2.1. General Overview of the Interventions

Psymate is a PDA-based EMA for symptom monitoring that aims to increase awareness about depression and the dynamics that characterize this disorder [62,63,64,67,68]. Psymate allows patients to record daily symptoms and affect. Based on these daily assessments, patients meet a clinician weekly and receive graphical feedback on the association between PA levels and daily life activities, events, or social interactions, as well as on the association between PA changes and the number of depressive complaints. In this way, patients have the chance to reflect on their affective state and the relationship between symptoms and contextual variables with a professional. According to Heron’s definition, “the key feature of all EMIs is that the treatment is provided to people during their everyday lives (i.e., in real time) and settings (i.e., real world)” [22]. Therefore, Psymate does not meet all the criteria for an EMI, as EMA-feedbacks are provided during weekly face-to-face sessions. However, we decided to include this intervention because we think it provides important insights about the potential of self-monitoring EMA as a therapeutic tool.

Likewise, Mobylize! constitutes an ecological intervention composed of a mobile application, an interactive website, and a system for email/telephone support [61]. The most innovative aspect of this application is the integration of self-reports with data from smartphone sensors. Mobylize! is provided with a context-aware system. Thanks to a machine learning algorithm, the application can predict the state of the patient (mood, emotions, cognitive/motivational states, activities, environmental context, and social context). Specifically, the system works in three different phases: (1) Data collection, during which 38 sensors collect sensor information; (2) learners, during which prompted self-reports are matched and paired with simultaneously labelled state data to develop predictive models; and (3) action components, a continuous process that analyses sensor data in order to update previous predictive models without the direct input of the user. Mobylize! is designed to prompt patients to assess mood, intensity of emotions, fatigue, pleasure, accomplishment, concentration, engagement, perceived control, location, and interactions five or more times a day. To accommodate new data, every new self-report is subsequently associated with the generation and modification of previous models. Thanks to this complex system, the mobile application sends tailored feedback to participants. Through the website, users can graphically visualize self-report patterns, read theoretical lessons, and use interactive tools, such as tailored plans and calendars, for monitoring daily activities. Lastly, a trained clinician contacts users periodically by phone or email to provide technical support, reinforce adherence, and enhance motivation. 

Help4Mood is a web-platform to self-monitor daily symptoms, mood, activities, and thoughts [66]. Based on a Cognitive Behavioural Therapy (CBT) approach, Help4Mood helps patients to reflect on the emotional and cognitive patterns related to depression. In addition to collecting daily self-reports, the application receives data from an actimetry sensor and acoustic analysis of speech. The innovative aspect of Help4Mood is the use of a virtual agent, completely customizable in terms of voice, clothing style, sex, and language, that communicates with users to provide tailored exercises and activities and guide them through the daily questionnaires. The application also has an emergency section called the “crisis plan”: As soon as symptom worsening is detected, the application prompts users to contact a professional or a relative. 

Finally, Medlink is a mobile application to support and monitor MDD patients taking antidepressant medication [65]. The main purpose of the app is to address the failure points that usually occur between professionals and newly diagnosed patients. On the one hand, the application provides users with weekly psychoeducation material and sends suggestions about medication management and how to deal with depressive symptoms. On the other hand, it monitors patients’ treatment and depressive symptoms. Every four weeks, personal communication with a professional is scheduled to give patients monthly feedback about disease progression.

#### 3.2.2. Effectiveness of the Intervention

Psymate was tested in a sample of 102 clinically depressed patients in a three-arm randomized controlled trial [62,63,64,67] with an experimental condition (treatment as usual – TAU - and six-week Psymate treatment, with weekly face-to-face feedback sessions), a pseudo-experimental condition (TAU and Psymate without EMA face-to-face feedbacks), and a control condition (TAU). Three different categories of weekly feedback were provided: (1) Positive affect, (2) positive affect in relation to events appraised with an internal versus external locus of control, and (3) positive affect in relation to social interactions. Results showed a significant reduction in depressive symptoms in the experimental group that was maintained in the follow-up assessment. Participants in the pseudo-experimental condition reported decreased depressive symptoms in the first weeks of the treatment, but this gain was not maintained across the weeks. Notably, the use of Psymate was associated with increased levels of perceived empowerment, regardless of the presence of weekly feedback, and with increased experienced PA throughout the treatment. Decreased depressive symptoms were also associated with increased positive daily behaviours. Finally, Widdershoven and colleagues observed a significant improvement in negative emotions’ differentiation and a close-to-significance improvement in positive emotions’ differentiation after 6-weeks of self-monitoring, regardless of EMA-derived feedbacks [68].

Mobylize! was tested in a small pilot study with a sample of 7 MMD patients [61]. According to the results, the use of Mobylize! significantly reduced depressive symptoms, both on a self-rated measure (PHQ-9) and a clinician-based evaluation (Quick Inventory of Depressive Symptomatology-Clinician Rating, QUIDS-C), as well as anxiety symptoms, measured with the Generalized Anxiety Disorder Scale (GAD-7). At the end of the treatment, participants were also less likely to meet MDD diagnostic criteria. Nevertheless, the accuracy of the predictive model was low, especially for mood; higher accuracy was achieved by models that predicted location, conversational state, and social interactions (accuracy between 60% and 90%).

A randomized controlled trial was conducted to evaluate Help4Mood [66]. Twenty-eight depressed patients were recruited and randomized into two treatment groups: Help4Mood and TAU. Outcome measures, which included the Beck Depression Inventory (BDI) and Quick Inventory of Depressive Symptomatology—Self Report (QIDS-SR), indicated reduced symptoms in both samples. Nevertheless, patients in the TAU group achieved greater clinical improvement compared to patients who used the application. Notably, regular users were more likely to obtain greater clinical improvement compared to users with low compliance.

Finally, a preliminary study tested the efficacy of Medlink with 8 MDD patients [65]. On the one hand, medication monitoring showed promising outcomes. Patients reported taking 84% of their medication, which is significantly higher than medication adherence rates reported in the literature. On the other hand, depressive symptoms significantly decreased over the course of 4 weeks.

#### 3.2.3. Compliance and Dropout Rates

Regarding Psymate, the number of answered prompts in both the experimental and pseudo-experimental groups was 135.5 out of 180 (75.3%); participants completed 39.7 out of 50 pre-assessments (79.4%) and 23.7 out of 30 (79%) post-assessment observations. Moreover, 27 of the 33 participants (81.9%) allocated to the experimental group completed the intervention, whereas 32 out of 36 participants (88.89%) allocated to the pseudo-experimental group completed it. 

Throughout the 8-week treatment with Mobylize, the mean number of log-ins to the mobile application was 7.9 (approximately one per week), whereas the number of completed lessons on the website was 4.8 out of 9 (53.3%). The number of answered prompts drastically decreased throughout the treatment, from 15.3 in the first week to 4.8 in the last week, due to technical difficulties and connectivity problems. Seven out of eight participants (87.5%) completed the intervention: The only dropout was caused by technical problems with the smartphone.

Regarding Help4Mood, the authors indicated great variability in terms of time of use. Two participants used the application for one or two days, whereas three participants used it between 3 and 7 days. The remaining six participants used it more than 10 times, approximately twice a week. The mean use was 134 min. Eleven out of 13 (84.6%) participants completed the protocol and were assessed for the follow-up. One participant withdrew due to worsening mood.

Finally, participants entered the Medlink application approximately 17.4 times during the 4 weeks of data collection and answered 96% of the prompts. Seven out of nine users read the psychoeducation lessons from the first and second week, whereas only half of them read the third and fourth lessons. No dropouts were reported.

#### 3.2.4. Participants’ Feedback and Satisfaction

Using Likert scales ranging from 1 to 7, participants found that Psymate was very simple to use and provided clear instructions (verbal instructions = 6.6 ± 0.7; written instructions = 6.5 ± 1.0; Psymate answers = 2.6 ± 1.5). The number of daily prompts and the time needed to complete assessments was not stressful (number of beeps per day = 3.1 ± 1.6; time to answer = 2.5 ± 1.5). Finally, satisfaction with its most important feature, i.e., receiving EMA-derived feedback, indicated that the feedback was highly appreciated (usefulness of feedback = 6.2 ± 0.7) and considered valuable (feedback to improve daily skills = 5.4 ± 1.1). However, participants would have appreciated receiving more specific and practical advice related to the EMA-based feedback (3.2 ± 2.0). 

Regarding Mobylize, satisfaction with the application was rated as 5.71 on a scale from 1 to 7. Criticism was related to technical problems, such as loss of connectivity and subsequent failure to receive prompts. Interestingly, 86% of the participants reported that the intervention was particularly helpful for identifying NA triggers and avoiding distressing and maladaptive behaviours. Participants also suggested lengthening the intervention and adding more activities, such as a blog to talk with other users or a message service between patients and coaches. 

Participants involved in the Help4Mood study were quite satisfied with the application. Most of them would use it in everyday life and suggest it to other patients. The idea of a virtual agent to guide participants in completing the assessments was appreciated; however, some participants perceived the agent as too cold, repetitive, and not sufficiently realistic. Among the limitations, patients reported sometimes being bored by excessively long sessions. They would have appreciated receiving more psychoeducational material and a more tailored experience, allowing them to access their preferred materials and activities without restrictions. 

Medlink’s usability was assessed using 4 items from the Usefulness, Satisfaction, and Ease of Use Questionnaire (USE). On a scale from 1 to 7, participants reported encouraging scores for ease of use (mean = 5.7 ± 1.1) and learnability (mean = 6.1 ± 1.5), but low scores for perceived usefulness (mean = 4.6 ± 1.0) and satisfaction (mean = 4.8 ± 0.8). Furthermore, encouraging ratings were observed for the weekly psychoeducation lessons (liking = 6.0 ± 1.1; ease of use = 6.6 ± 0.5; learnability = 6.6 ± 0.5; and usefulness = 5.8 ± 1.7), which were also reported to be the most interesting and useful parts of the application. Finally, feedback interviews showed neutral comments regarding daily self-reports, that were perceived as not very useful; contrasting opinions were collected regarding feedback graphs.

## 4. Discussion

To date, the scientific literature has mostly been based on studies conducted in laboratory settings, thus understudying the daily dynamics of psychopathology [70]. Therefore, unobtrusively monitoring behavioural (i.e., sensors), physiological (i.e., biosensors), and cognitive/emotional (i.e., self-reports) factors in ecological settings collected through portable and wearable devices can provide new information about elusive psychological constructs that are usually defined by the complex dynamics of contexts and variability. Accordingly, the research field could benefit from the use of novel technologies to better explore MDD mechanisms and delineate new theoretical models based on ecological observations. 

Compared to paper and pencil daily diaries, the use of electronic devices, and especially smartphones, could further increase the six EMA advantages identified by Ebner-Premier (Table 5) [16]: (a) The automation of the entire process directly on a mobile device, such as a smartphone, can provide greater control over backfilling and higher temporal precision in the administration, planning, and randomization of prompts; (b) the use of ICTs can offer additional possibilities for multimodal assessments, with data supplied by embedded sensors and wearable unobstructed biosensors that can automatically be coordinated with the collection of self-reports; (c) the use of mobile devices reduces the effort required of users in completing daily assessments and prevents errors by researchers and clinicians due to manual data entry; (d) smartphones offer the possibility of providing real-time EMA-derived feedback that can be an important therapeutic tool for patients’ self-monitoring, in addition to the possibility of sending real-time alerts to clinicians in case of need. In this regard, smartphones have the potential of becoming global low-cost tools that can also be adopted in the clinical field. Currently, 2.32 billion people in the world use smartphones, and it has been estimated that, by 2020, 70% of the world’s population will own one [71]. The potential of these devices is also supported by the evidence showing that people with serious mental and physical illnesses own and regularly use smartphones [72] and are interested in using applications for their health [26].

As pointed out in this review, the widespread adoption of EMA for the investigation of depression has led to novel insights into different aspects of the disease, including emotion reactivity, cortisol patterns, or daily rumination. We discussed different sampling methods that can be used in EMA protocols, showing that the signal-contingent design with prompt randomization or semi-randomization is the most widely adopted option when dealing with variables, such as affect and symptom monitoring. We also reported compliance and dropout rates, which showed encouraging results, with most of the studies reporting more than 70% adherence. Nevertheless, the gap between clinical practice and research is still quite wide, as revealed by the low number of studies that adopt this approach to assess and monitor patients for clinical purposes or implement EMA in clinical settings. Accordingly, many issues still need to be addressed. To date, no standard and validated sets of items have been developed for EMA protocols, raising the problem of context validity. Moreover, further research should be conducted to improve patients’ compliance and reduce dropout. Due to the intrinsic nature of the disease, depressed patients could be less likely to consistently complete daily assessments. In a previous study, we observed that compliance was higher in EMA administered through a smartphone and when patients were prompted less than 8 times a day [73]. However, a meta-analysis should be conducted to more precisely identify the factors that improve adherence (see, for example, [74]), thus providing some sort of guideline for the design of EMA. Indeed, we strongly believe that clinical practice could benefit from the use of EMAs for several reasons. First, EMAs can be useful for diagnostic purposes. Traditional diagnostic procedures usually involve a static moment in time, including semi-structured interviews (e.g., Mini-International Neuropsychiatric Interview) complemented by self-report measures. However, ample evidence shows the dynamic nature of affective states and mood [75]. Furthermore, these dynamics greatly vary from person to person, reasons for which ideographic approaches may shed light upon the structure of individual symptom dynamics [60]. Consequently, by means of EMAs, a more accurate diagnostic process could be pursued. Likewise, the continuous monitoring of patients’ symptoms would allow clinicians to monitor the efficacy of a treatment over time [76], predict short-term mood changes [77], detect symptoms’ worsening in an early stage [78], and create continuous communication between clinicians and patients. On the other hand, the use of daily mood and symptom self-ratings could provide more ecological assessments, overcoming recall bias and capturing the dynamics of human functioning in daily life that cannot be detected with traditional tools. 

Our results also highlight the existence of a small number of EMIs for depression. In the current literature, only four ecological interventions have been developed, and only two of them were tested in a randomized-controlled trial (RCT). Our review showed promising results in terms of patient satisfaction and clinical efficacy, further supporting the need for more efforts in this direction. However, compliance rates were sometimes not encouraging, and a major challenge is to encourage regular use of these technologies throughout the entire treatment process [79]. Accordingly, future research should focus on the concept of users’ motivation and engagement, taking into consideration the adoption of focus groups with patients during treatments, using mixed quantitative and qualitative designs to obtain as much information as possible to guide future developments, and extending the effects of gamification features on adherence and compliance [80]. In other words, greater attention should be paid to the needs and characteristics of the target population. Considering feedback from users, here, we were able to identify three EMI features that were highly appreciated: The possibility of receiving visual feedback about daily assessments and, therefore, self-monitoring of daily patterns; the availability of psychoeducational material on depression and its mechanisms; and the opportunity to have continuous or periodic communication with a trained clinician. 

In this review, we found that most of the EMAs were based only on self-reports, whereas more attempts to integrate this information with data gathered from sensors and biosensors were observed for EMIs. Recent advances in sensor technologies have had an impact on applications for remote health [81], such as postoperative recovery [82], treatment for chronic patients [83], and monitoring of elderly individuals [84]. Consistently, the hierarchical sensing model proposed by Mohr highlights the great revolution that new sensors and biosensors can bring to the field of mental health [85], making it possible to collect raw sensor data (i.e., the lower level of the hierarchy) that can be converted into “behavioural markers” through machine learning and data mining methods [18]. 

Smartphone sensors further increase the potentially collectable information, allowing the reconstruction of people’s habits, sleep patterns, or social life by using embedded sensors, such as accelerometers, calls, short message service (SMS), social network data, or geolocation. In other words, it is now possible to infer and collect behavioural information without necessarily asking the person to report it.

Even though they were not investigated in the studies targeted at MDD patients discussed here, several opportunities can be found in the integration of EMA and EMI platforms with behavioural and physiological signal processing, further mediated by machine learning algorithms. On the one hand, several behavioural signals are readily collectable with the use of smartphone sensors, even though they may lack the required specificity for mood recognition and prediction, as found by the Mobylize! study [61]. On the other hand, due to recent advancements in sensor technologies, physiological signals can be nowadays recorded unobtrusively by means of, for example, smartwatches and chest bands. These could provide an EMA and/or EMI platform with additional markers that more closely correlate to a person’s affective state, and that can be used as input to the analysis performed [61]. Consistently, models can be automatically learned that continuously estimate the patient’s affective state by extracting and analysing salient features of physiological signals [86]. For instance, electrodermal activity (EDA) and heart rate variability (HRV) have been extensively investigated as correlates of users’ affective state, and they are considered non-invasive. They do not involve recording sensitive information (as opposed to, for example, cameras and acoustic signals), and associated sensors do not interfere with users’ daily routines. Consistently, patient-specific models can be automatically learned that continuously estimate the patient’s affective state by extracting and analysing salient features of physiological signals [86]. 

Unfortunately, the relation between physiological signals and affective states is not trivial and mixed results are discussed in the literature [87]. Building on recent advances of machine learning, recent studies obtained promising results by means of model personalisation for stress recognition [88] and deep learning for mood prediction [89] using a combination of behavioural and physiological markers in non-clinical populations. If thoroughly tested and consolidated through experimental validations in EMA settings, a model of this type could provide a finer-grained description of the evolution of the patient’s disorder throughout a long-term study, compared to surveys that are usually filled in just a few times a day. It can be considered less obstructive to the patient’s life because physiological data are recorded passively and do not require extra effort from the patient. Furthermore, in EMI settings, if the recognition algorithm detects that the patient is in a critical state, it can automatically trigger an intervention module associated with the platform or open a communication channel between the patient and his/her therapist. Alternatively, predictive models that combine information from physiological and behavioural signals to estimate the patient’s future mood, stress level, and self-reported health (one or a few days in advance) can be automatically inferred [89]. After identifying a risk threshold, these models would make it possible to plan interventions (or involve the therapist) in advance, that is, before the patient’s affective state reaches a critical state. 

We should, however, recognize that the use of EMAs and EMIs has some limitations. These approaches are time-consuming and may be perceived as invasive by users. Patients are required to complete multiple assessments throughout a day, and protocols often last weeks. Moreover, people might not be willing to share personal information. Finally, in terms of more ecological validity, they may be advantageous for clinical purposes, but disadvantageous for research aims, because they imply less experimental control. Because the data are collected during everyday life and in naturalistic environments, it becomes hard or even impossible to have complete control over the setting, and, therefore, it is not possible to rule out the role of confounding variables. Nevertheless, due to the implementation of novel statistical procedures, a balance between research necessities and clinical utility could be achieved [90]. If this were the case in the near future, EMAs and EMIs would undoubtedly transform the field of mental health, greatly contributing to the bridging of science and practice [91,92].

Overall, this systematic review clearly shows the emergence of ecological assessment and intervention as a promising avenue for clinical psychology. The focus of the review was limited to a specific clinical population. Still, promising results have been already shown also regarding the application of EMA and EMI to anxiety disorders [93,94] and stress-related disorders [95,96], highlighting the potential of these tools to provide psychological support in daily life and to investigate symptom fluctuations across time. However, similar limitations and burning issues were also evidenced, including the need for more high-quality trials, the gap between the clinical and research field, and the importance of making EMAs and EMIs as engaging and tailored as possible. Altogether, there is evidence showing the feasibility and preliminary efficacy of these approaches, but much more research should be conducted before drawing definite conclusions.

## Figures and Tables

**Table 1:** Detailed search strategy.

Process	Results
PubMed/Medline	2758
Web of Science	2235
Total	4993
Not duplicated	3613
Excluded (after reading title and abstract)	3212
Retrieved	401
Excluded (after applying inclusion criteria)	361
Excluded (missing experimental data)	0
Final included articles	40

**Table 2:** More detailed information about the selected Ecological Momentary Assessment studies.

	Authors	Sample	Variables	Device(s)	Duration	Prompts Per Day	Sampling Schema	Compliance	Sensor(s)	Primary Outcome(s)
**Recall bias**	[8]	MDD (*n* = 26), and HCG (*n* = 25)	Affect	Palm Tungsten E2	7 days	8	Semi-randomized	89%	No	Both depressed and non-depressed participants overestimate the retrospective recall of PA and NA. Depressed patients are more inaccurate in recalling NA.
	[26]	MDD (*n* = 13)	Randomized items from PHQ-9 questionnaire	“Mindful Moods” mobile application	29/30 days	3	Randomized	78%	No	Even if strongly correlated, the PHQ-9 scores collected through the mobile application are significantly higher than those obtained though the retrospective paper-and-pencil PHQ-9.
**Symptoms monitoring**	[39]	MDD (*n* = 20), and BD (*n* = 21)	Affect; stressors; behaviours; environment; social context	PDA	3 days	5	Fixed sampling scheme	85.7%	No	High rates of acceptance and compliance are observed among both samples. Participants show a practice effect, i.e., faster responses over the course of the study.
	[40]	MDD (*n* = 26)	Depressive and anxiety symptoms	Palm Treo 650 Smartphone (Mental Health Telemetry mobile application)	14 days	1	Selected by the patient	Not specified	No	Self-reported ratings of improvement at day 7 predict response to the treatment.
	[41]	MDD (*n* = 59)	Symptoms, sleep patterns, cognitive functioning	iHOPE smartphone application	8 weeks	2 (symptoms)	Not specified	Not specified	No	Baseline depression scores evaluated with HAM-D are associated with scores of PHQ-9, VAS for depression and anxiety symptoms collected with the application.
						1 (sleep duration and quality)				
**Cortisol**	[42]	MDD (*n* = 32), mD (*n* = 18), and HCG (*n* = 50)	Daily activities (frequency, social contacts); cortisol	Palm Pilot M100	4 days (over a maximum period of 7 days)	4 (saliva samples)	Fixed sampling scheme	Not specified	No	In the control sample, daily activities are negatively associated with cortisol levels. This association is not observed in depressed patients.
						1 (self-report)				
	[43]	MDD (*n* = 37), and HCG (*n* = 36)	Sleep patterns; social contacts; cortisol	Palm Pilot M100	3 non-consecutive days (over a maximum period of 7 days)	3 (saliva samples)	Fixed sampling scheme	93%	No	Depressed patients show lower cortisol awakening response, lower sleep quality, and more negative social interactions.
						1 (self-report)				
	[31]	MDD (*n* = 46) and HCG (*n* = 19)	Physiological indices (HR, respiration, accelerometer, cortisol); mood	LifeShirt System, with an integrated hand-held computer	1 day	6 (self-reports)	Fixed sampling scheme	91%	LifeShirt System (HR, respiration, actigraphy)	Cortisol level and HRV do not differ between the two groups. Interestingly, NA is negatively correlated with HRV only in the control sample.
						5 (saliva samples)				
	[44]	Remitted MDD (*n* = 31) and HCG (*n* = 32)	Mood; ruminative self-focus; stressful events; cortisol	Palm Tungsten E2	2 consecutive days	10	Semi-randomized	94%	No	Rumination and low mood are associated with increased activation of the HPAA. In remitted patients, HPAA is less responsive to subtle emotional events.
	[36]	MDD (*n* = 15), and HCG (*n* = 15)	Affect; cognition; daily activities; cortisol	PsyMate	30 days	3	Fixed sampling scheme	92.5%	ActiCal (Respironics, Bend, OR, USA)	Compared to healthy participants, depressed patients report higher cortisol levels, higher α-amylase levels, and a greater ratio of α-amylase over cortisol. This latter association, however, disappears when correction for lifestyle factors is applied.
	[45]	MDD (*n* = 15), and HCG (*n* = 15)	Affect; cortisol	PsyMate	30 days	3	Fixed sampling scheme	92.5%	No	PA and NA are bidirectionally associated with cortisol levels. Nevertheless, the direction, sign, and timing of this association show great variability among subjects.
**Sleep patterns**	[46]	MDD (*n* = 35), mD (*n* = 25), and HCG (*n* = 36)	Positive and negative affects	PDA	3 days	10	Semi-randomized	65%	No	Sleep quality predicts lower PA, but not NA. Low PA is associated with poor subjective sleep quality and self-reported daily dysfunction.
	[47]	MDD and mD (*n =* 60), and HCG (*n =* 35)	Positive and negative affects; events appraisal	Palm Pilot Zire 22	3 days	10	Semi-randomized	65%	No	In the non-clinical sample, sleep disturbances are associated with enhanced NA in response to negative events. Considering depressed patients, sleep disturbances negatively influence the emotional reactivity to both neutral and negative events.
	[48]	MDD (*n =* 27), and HCG (*n =* 27)	PA and NA, sleep quality; tiredness; rumination	PsyMate	30 days	3	Fixed sampling scheme	96%	No	Sleep quality directly influences PA and NA experienced during the following day, but not vice versa. Tiredness is a mediator.
	[49]	MDD (*n =* 14) and HCG (*n =* 15)	PA; NA; fatigue; sleep; activities; cognition; melatonin	PsyMate	30 days	3	Fixed sampling scheme	93%	No	Melatonin is associated with changes in affect and fatigue. However, changes in affect and fatigue are also predictors of melatonin levels. Individuals that do not show this association report higher depression severity and worse sleep quality.
	[38]	MDD (*n =* 27), and HCG (*n =* 27)	Sleep patterns	PsyMate	30 days	3	Fixed sampling scheme	96%	ActiCal (Respironics, Bend, OR, USA)	Sleep duration affects next-day physical activity. Depression does not moderate this association.
	[33]	MDD (*n =* 51)	Sleep patterns and quality; suicide ideation; entrapment perception	PRO-Diary actigraph watch (CamNtech)	7 days	6	Semi-randomized	89%	Accelerometer	Poor sleep quality, both objectively and subjectively evaluated, is associated with higher next-day suicide ideations. Suicide ideation does not influence sleep patterns and quality.
**Physical activity**	[50]	MDD (*n =* 53), and HCG (*n =* 53)	Physical activity; positive and negative affects	Palm Pilot Zire 22	7 days	8	Randomized	75%	No	Both samples show higher PA following physical activity. More specifically, depressed patients show a significantly higher increase in experienced PA levels after physical activity.
	[32]	MDD (*n =* 14) and HCG (*n =* 43)	Mood; physical symptoms; physical activity	Ruputer ECOLOG	Average: 37.43 days (range:18–67 days)	4	Semi-randomized	93%	Ambulatory Monitors Inc.—actigraph	Depressive mood is associated with increased intermittency of locomotor activity.
	[37]	MDD (*n =* 10), and HCG (*n =* 10)	Mood; cognition; daily activities; physical activity	PsyMate	30 days	3	Fixed sampling scheme	91%	ActiCal Respironics—actigraph	Despite the observation of large interindividual differences, results show a positive effect of physical activity on PA in all participants.
**Rumination**	[51]	MDD (*n =* 6) and HCG (*n =* 7)	Context; mood; depressive symptoms; EEG (at baseline)	Palm Pilot and EEG (at baseline)	7 days	5	Not specified	Not specified	No	Lower activation of bilateral PFC predicts higher rates of rumination, whereas higher levels of self-esteem are associated with lower right PFC activity.
	[35]	MDD (*n =* 18) and HCG (*n =* 18)	Thoughts; disturbing events; feelings; possible influencing factors; feelings; HR	Electronic diary implemented on a smartphone	1 day	Not reported	Semi-randomized	Not specified	RS 800CX; Bodyguard2 (HR and HRV)	Depressed participants show higher rates of perseverative cognition, which are associated with lower HRV.
	[52]	MDD (*n =* 38), GAD (*n =* 36), MDD with GAD comorbidity (*n =* 38), and HCG (*n =* 33)	Events stressfulness; rumination	Palm Pilot Zire 22	7 days	8	Randomized	72%	No	MDD and GAD participants show the same level of rumination, which is even more severe in comorbid cases. Higher rates of rumination are predictive of worse affect, more maladaptive behaviours, and more severe symptoms.
	[53]	MDD (*n =* 16), GAD (*n =* 15), MDD with GAD comorbidity (*n =* 20), and HCG (*n =* 19)	Rumination; worry; PA and NA; significant events	Palm Pilot Zire 22	6 to7 days	8	Semi-randomized	65%	No	Levels of rumination among all the clinical samples are higher in response to significant events. Decreased PA and increased NA are associated with higher momentary rumination.
**Emotional reaction**	[54]	Remitted MDD (*n =* 55) and HCG (*n =* 55)	Perceived stress; mood	Hand held Psion “Revo” computer	7 days	5	Randomized	90%	No	Past episodes of depression are likely to increase the vulnerability to stressful events, especially in male participants.
	[55]	MDD (*n =* 35), mD (*n =* 26), and HCG (*n =* 38)	Context; mood; events (nature of the event; location; people involved; affective rating)	Palm Pilot Zire 22	3 non-consecutive days (over a period of 5 days)	10	Semi-randomized	65%	No	Both MDD and mD patients show lower levels of positive affect and rate events as more stressful and unpleasant than the control group. Furthermore, they show a higher reduction in negative feelings after positive events.
	[50]	MDD (*n =* 53), and HCG (*n =* 53)	Affect; significant events	Palm Pilot Zire 22	7/8 days	8	Randomized	78%	No	Results point out greater emotional instability with respect to NA in depressed patients. No differences are observed in terms of reactivity, inertia, and instability in PA.
	[56]	MDD (*n =* 21) and MDD with BPD comorbidity (*n =* 20)	Affect and mood; events; subjective affective reactivity	Smartphone to access a web platform	7 days	5	Randomized	94%	No	Comorbidity with BPD does not imply major affective instability, but it is associated with lower subjective perception of affective reactivity.
	[57]	Remitted MDD (*n =* 10) and HCG (*n =* 11)	Mood; PA and NA; visual mental imagery	“Imagine your Mood”, smartphone application	3 days a week, for 8 weeks	10	Semi-randomized	Not specified	No	In both samples, higher levels of visual imagery-based processing are associated with higher levels of PA and better mood, regardless of the valence of the imagery content. Elevated levels of visual imagery-based processing are not associated with daily affective reactivity.
	[58]	MDD (*n =* 51), and BPD (*n =* 80)	PA, NA, fear; hostility; sadness; interpersonal events	Palm Pilot Zire 31	28 days	6	Semi-randomized	86%	No	Rejection and disagreement increase NA (especially hostility and sadness) both at a momentary and daily level, regardless of the diagnosis. The association between rejection/disagreement and hostility is stronger in BPD patients.
	[59]	MDD (*n =* 12), DD (*n =* 3), BD (*n =* 15)	Affect; location; social context; gambling desire/motivation/activities	Palm Pilot Zire 22	30 days	3	Randomized	73%	No	High levels of sadness and arousal are predictive of gambling desire, regardless of the diagnosis. Depressed individuals are likely to gamble to increase PA or for social reasons.
	[60]	MDD (*n =* 15), GAD (*n =* 25)	Symptoms, PA, NA, rumination, behavioural avoidance, reassurance seeking	Web-based survey	30 days	4	Not specified	Not specified	No	Using a person-by-person approach, results show that moment-to-moment symptomatology is mainly driven by positive mood, hopelessness, anger, and irritability, but not depressed mood, anhedonia, or worry.

MDD: major depressive disorder; mD: minor depression; HCG: healthy control group; BDP: borderline personality disorder; GAD: generalized anxiety disorders; PDA: personal digital assistant; DD: dysthymic disorder; PFC: prefrontal cortex; PA: positive affect; NA: negative affect; HRV: heart rate variability; PHQ-9: Patient Health Questionnaire-9; QUID-SR: Quick Inventory of Depressive Symptomatology—Self Report; HADS-A: Hospital Anxiety and Depression Scale; PANAS: Positive and Negative Affect Schedule; HPAA: hypothalamic-pituitary-adrenal axis.

**Table 3:** More detailed information about the selected Ecological Momentary Intervention studies.

Authors	Name	Sample	Intervention	Duration	Prompts	Sampling Schema	Sensor(s)	Primary Outcome(s)
[61]	Mobylize!	MDD (*n =* 7), with different comorbidities	Mobylize! is a context-aware system, composed of three main elements: (1) A mobile application for the collection of self-reports; (2) a website with feedback and theoretical lessons; (3) periodic contacts with trained coaches	8 weeks	5/day	Randomized	38 concurrent sensors integrated in the phone	Mobylize! significantly reduced depressive symptoms. Predictive models did not reach high levels of accuracy, especially for mood.
[62]	PsyMate	MDD (*n =* 102): Experimental condition (*n =* 33), pseudo-experimental condition (*n =* 36), control condition (*n =* 33)	Daily assessment of self-reports and weekly EMA-derived feedback through face-to-face sessions	3 days per week, for 6 weeks	10/day	Semi-randomized	No	The use of EMA-derived feedback as a complementary intervention to pharmacological treatment significantly decreased depressive symptoms. These improvements were also maintained over time.
[63]	PsyMate	MDD (*n =* 102): Experimental condition (*n =* 33), pseudo-experimental condition (*n =* 36), control condition (*n =* 33)	Daily assessment of self-reports and weekly EMA-derived feedback through face-to-face sessions	3 days per week, for 6 weeks	10/day	Semi-randomized	No	The use of Psymate as a technique of self-monitoring could improve patients’ feelings of empowerment.
[64]	PsyMate	MDD (*n =* 102): Experimental condition (*n =* 33), pseudo-experimental condition (*n =* 36), control condition (*n =* 33)	Daily assessment of self-reports and weekly EMA-derived feedback through face-to-face sessions	3 days per week, for 6 weeks	10/day	Semi-randomized	No	Face-to-face EMA-derived feedback sessions did not increase the rate of PA experienced during or shortly after the intervention.
[65]	Medlink	MDD (*n =* 8)	Medlink is a mobile application delivering psychological support to MDD patients. The application provides users with: (1) psychoeducation; (2) weekly symptom assessment; (3) medication adherence monitoring; (4) monthly communication with a professional based on the previous points	4 weeks	1/week	Not specified	No	Medlink was positively evaluated by participants, especially regarding the weekly psychoeducation lessons. Depression severity of participants significantly decreased over the course of the experiment.
[66]	Help4Mood	MDD (*n =* 28): Experimental condition (*n =* 14) and control condition (*n =* 14)	Web platform providing daily assessment of symptoms, self-monitoring, and tailored activities. The delivered content is created in response to the user’s actions through a virtual agent	About 5 weeks	CESD- VAS-VA: 1/day	Not specified	Accelerometer and acoustic speech analysis	Only half of the participants used Help4Mood regularly. Significant changes in depressive symptoms were observed only among regular users.
					PHQ-9: 1/week			
[67]	PsyMate	MDD (*n =* 102): Experimental condition (*n =* 33), pseudo-experimental condition (*n =* 36), control condition (*n =* 33)	Daily assessment of self-reports and weekly EMA-derived feedback through face-to-face sessions	3 days per week, for 6 weeks	10/day	Semi-randomized	No	The use of EMA-derived feedback decreased depressive symptoms and improved maladaptive behaviours.
[68]	PsyMate	MDD (*n =* 79): Experimental condition (*n =* 25), pseudo-experimental condition (*n =* 30), control condition (*n =* 24)	Daily assessment of self-reports and weekly EMA-derived feedback through face-to-face sessions	3 days per week, for 6 weeks	10/day	Semi-randomized	No	The use of a self-monitoring EMA improves negative emotion differentiation.

MDD: major depressive disorder; PA: positive affect; CESD-VAS-VA: brief visual analogue scale version of the Center of Epidemiological Studies Depression. Scale; PHQ-9: Patient Health Questionnaire.

**Table 4:** Fields of application of EMAs for Major Depressive Disorder.

Field of Application	Retrieved Articles	Aim	Advantages
Recall bias	[8,26]	Experimental	No retrospective bias; control over backfilling; repeated momentary measurements.
Symptoms monitoring	[39,40,41]	Clinical	Continuous monitoring (symptoms assessment, treatment progress); real-time feedback to clinicians (e.g., crisis plan) and users (e.g., patterns visualization).
Cortisol dysregulation	[31,36,42,43,44,45]	Experimental	Role of contextual variables; temporal relationship between physiological measures and self-reports.
Sleep patterns	[33,38,46,47,48,49]	Experimental	Control over backfilling; no retrospective bias; integration of self-reports with passive data supplied by sensors and biosensors.
Physical activity	[32,37,50]	Experimental	Role of contextual variables; integration of self-reports with passive data supplied by sensors; temporal relationship between physiological measures and self-reports.
Rumination	[35,51,52,53]	Experimental	Role of contextual variables; rumination deployment across time.
Affect and emotional reactivity	[50,54,55,56,57,58,59,60],	Experimental	Role of contextual variables; temporal deployment of affect and emotional reactivity.

**Table 5:** Benefits of using EMA for mood dysregulation and mood disorders as described by Ebner-Premier [16].

	Advantages	Implications
1	Real-time assessments	Reduction in retrospective bias and increase in accuracy.
2	Repeated measurements	Better comprehension of time-dependent processes and dynamic changes in symptoms.
3	Multimodal assessments	Contemporary analysis of behaviours, physiological signals, and subjective experiences.
4	Context-specific information	Assessment of symptoms as context-dependent.
5	Interactive assessments	Real-time customizable and interactive feedback.
6	Generalizability	Higher ecological validity and collection of more representative data.

## References

[1] World Health Organization (2017). Depression and Other Common Mental Disorders: Global Health Estimates.

[2] Katon W., Ciechanowski P. (2002). Impact of major depression on chronic medical illness. J. Psychosom. Res..

[3] Simon G.E. (2003). Social and economic burden of mood disorders. Biol. Psychiatry.

[4] Sullivan L.E., Fiellin D.A., O’Connor P.G. (2005). The prevalence and impact of alcohol problems in major depression: A systematic review. Am. J. Med..

[5] Swendsen J.D., Merikangas K.R. (2000). The comorbidity of depression and substance use disorders. Clin. Psychol. Rev..

[6] McConville C., Cooper C. (1996). Mood variability and the intensity of depressive states. Curr. Psychol..

[7] Peeters F., Berkhof J., Delespaul P., Rottenberg J., Nicolson N.A. (2006). Diurnal mood variation in major depressive disorder. Emotion.

[8] Ben-Zeev D., Young M.A., Madsen J.W. (2009). Retrospective recall of affect in clinically depressed individuals and controls. Cogn. Emot..

[9] Chamberlain S.R., Sakakian B.J. (2006). The neuropsychology of mood disorders. Curr. Psychiatry Rep..

[10] Möller H.J., von Zerssen D. (1995). Self-rating procedures in the evaluation of antidepressants: Review of the literature and results of our studies. Psychopathology.

[11] Gotlib I.H., Joormann J. (2010). Cognition and Depression: Current Status and Future Directions. Annu. Rev. Clin. Psychol..

[12] Csikszentmihalyi M., Larson R. (1987). Validity and Reliability of the Experience- Sampling Method. J. Nerv. Ment. Dis..

[13] Shiffman S., Stone A.A., Hufford M.R. (2008). Ecological Momentary Assessment. Annu. Rev. Clin. Psychol..

[14] Stone A.A., Shiffman S., Atienza A.A., Nebeling A. (2007). Historical roots and rationale of ecological momentary assessment (EMA). The Science of Real-Time Data Capture: Self-Reports in Health Research.

[15] Aan het Rot M., Hogenelst K., Schoevers R.A. (2012). Mood disorders in everyday life: A systematic review of experience sampling and ecological momentary assessment studies. Clin. Psychol. Rev..

[16] Ebner-Priemer U.W., Trull T.J. (2009). Ecological momentary assessment of mood disorders and mood dysregulation. Psychol. Assess..

[17] Marzano L., Bardill A., Fields B., Herd K., Veale D., Grey N., Moran P. (2015). The application of mHealth to mental health: Opportunities and challenges. Lancet Psychiatry.

[18] Mohr D.C., Zhang M., Schueller S.M. (2017). Personal Sensing: Understanding Mental Health Using Ubiquitous Sensors and Machine Learning. Annu. Rev. Clin. Psychol..

[19] van de Ven P., O’Brien H., Henriques R., Klein M., Msetfi R., Nelson J., Rocha A., Ruwaard J., O’Sullivan D., Riper H. (2017). ULTEMAT: A mobile framework for smart ecological momentary assessments and interventions. Internet Interv..

[20] Henderson C., Evans-Lacko S., Thornicroft G. (2013). Mental illness stigma, help seeking, and public health programs. Am. J. Public Health.

[21] Kazdin A.E., Blase S.L. (2011). Rebooting Psychotherapy Research and Practice to Reduce the Burden of Mental Illness. Perspect. Psychol. Sci..

[22] Heron K.E., Smyth J.M. (2010). Ecological momentary interventions: Incorporating mobile technology into psychosocial and health behaviour treatments. Br. J. Health Psychol..

[23] Donker T., Petrie K., Proudfoot J., Clarke J., Birch M.R., Christensen H. (2013). Smartphones for smarter delivery of mental health programs: A systematic review. J. Med. Internet Res..

[24] Cuijpers P., Donker T., Van Straten A., Li J., Andersson G. (2010). Is guided self-help as effective as face-to-face psychotherapy for depression and anxiety disorders? A systematic review and meta-analysis of comparative outcome studies. Psychol. Med..

[25] Asada H.H., Shaltis P., Reisner A., Rhee S., Hutchinson R.C. (2003). Mobile Monitoring with Wearable Photoplethysmographic Biosensors. IEEE Eng. Med. Biol. Mag..

[26] Torous J., Friedman R., Keshvan M. (2014). Smartphone ownership and interest in mobile applications to monitor symptoms of mental health conditions. J. Med. Internet Res..

[27] Moher D., Liberati A., Tetzlaff J., Altman D.G., PRISMA Group (2009). Preferred reporting items for systematic reviews and meta analyses: The Prisma Statement. PLoS Med..

[28] Colombo D., Palacios A.G., Alvarez J.F., Patané A., Semonella M., Cipresso P., Kwiatkowska M., Riva G., Botella C. (2018). Current state and future directions of technology-based ecological momentary assessments and interventions for major depressive disorder: Protocol for a systematic review. Syst. Rev..

[29] Delgadillo J., de Jong K., Lucock M., Lutz W., Rubel J., Gilbody S., Ali S., Aguirre E., Appleton M., Nevin J. (2018). Feedback-informed treatment versus usual psychological treatment for depression and anxiety: A multisite, open-label, cluster randomised controlled trial. Lancet Psychiatry.

[30] Downs S.H., Black N. (1998). The feasibility of creating a checklist for the assessment of the methodological quality both of randomised and non-randomised studies of health care interventions. J. Epidemiol. Community Health.

[31] Conrad A., Wilhelm F.H., Roth W.T., Spiegel D., Taylor C.B. (2008). Circadian affective, cardiopulmonary, and cortisol variability in depressed and nondepressed individuals at risk for cardiovascular disease. J. Psychiatr. Res..

[32] Kim J., Nakamura T., Kikuchi H., Yoshiuchi K., Sasaki T., Yamamoto Y. (2015). Covariation of Depressive Mood and Spontaneous Physical Activity in Major Depressive Disorder: Toward Continuous Monitoring of Depressive Mood. IEEE J. Biomed. Heal. Inform..

[33] Littlewood D.L., Kyle S.D., Carter L.-A., Peters S., Pratt D., Gooding P. (2018). Short sleep duration and poor sleep quality predict next-day suicidal ideation: An ecological momentary assessment study. Psychol. Med..

[34] Adams Z.W., McClure E.A., Gray K.M., Danielson C.K., Treiber F.A., Ruggiero K.J. (2017). Mobile devices for the remote acquisition of physiological and behavioral biomarkers in psychiatric clinical research. J. Psychiatr. Res..

[35] Ottaviani C., Shahabi L., Tarvainen M., Cook I., Abrams M., Shapiro D. (2015). Cognitive, behavioral, and autonomic correlates of mind wandering and perseverative cognition in major depression. Front. Neurosci..

[36] Booij S.H., Bos E.H., Bouwmans M.E.J., Van Faassen M., Kema I.P., Oldehinkel A.J., De Jonge P. (2015). Cortisol and α-amylase secretion patterns between and within depressed and non-depressed individuals. PLoS ONE.

[37] Stavrakakis N., Booij S.H., Roest A.M., de Jonge P., Oldehinkel A.J., Bos E.H. (2015). Temporal dynamics of physical activity and affect in depressed and nondepressed individuals. Health Psychol..

[38] Bouwmans M.E.J., Oude Oosterik N.A.M., Bos E.H., de Groot I.W., Oldehinkel A.J., de Jonge P. (2018). The Temporal Order of Changes in Physical Activity and Subjective Sleep in Depressed Versus Nondepressed Individuals: Findings From the MOOVD Study. Behav. Sleep Med..

[39] Husky M.M., Gindre C., Mazure C.M., Brebant C., Nolen-Hoeksema S., Sanacora G., Swendsen J. (2010). Computerized ambulatory monitoring in mood disorders: Feasibility, compliance, and reactivity. Psychiatry Res..

[40] Schaffer A., Kreindler D., Reis C., Levitt A.J. (2013). Use of Mental Health Telemetry to Enhance Identification and Predictive Value of Early Changes During Augmentation Treatment of Major Depression. J. Clin. Psychopharmacol..

[41] Hung S., Li M.-S., Chen Y.-L., Chiang J.-H., Chen Y.-Y., Hung G.C.-L. (2016). Smartphone-based ecological momentary assessment for Chinese patients with depression: An exploratory study in Taiwan. Asian J. Psychiatr..

[42] Stetler C., Dickerson S.S., Miller G.E. (2004). Uncoupling of social zeitgebers and diurnal cortisol secretion in clinical depression. Psychoneuroendocrinology.

[43] Stetler C., Miller G.E. (2005). Blunted cortisol response to awakening in mild to moderate depression: Regulatory influences of sleep patterns and social contacts. J. Abnorm. Psychol..

[44] Huffziger S., Ebner-Priemer U., Zamoscik V., Reinhard I., Kirsch P., Kuehner C. (2013). Effects of mood and rumination on cortisol levels in daily life: An ambulatory assessment study in remitted depressed patients and healthy controls. Psychoneuroendocrinology.

[45] Booij S.H., Bos E.H., de Jonge P., Oldehinkel A.J. (2016). The temporal dynamics of cortisol and affective states in depressed and non-depressed individuals. Psychoneuroendocrinology.

[46] Bower B., Bylsma L.M., Morris B.H., Rottenberg J. (2010). Poor reported sleep quality predicts low positive affect in daily life among healthy and mood-disordered persons: Sleep quality and positive affect. J. Sleep Res..

[47] O’Leary K., Small B.J., Panaite V., Bylsma L.M., Rottenberg J. (2017). Sleep quality in healthy and mood-disordered persons predicts daily life emotional reactivity. Cogn. Emot..

[48] Bouwmans M.E.J., Bos E.H., Hoenders H.J.R., Oldehinkel A.J., de Jonge P. (2017). Sleep quality predicts positive and negative affect but not vice versa. An electronic diary study in depressed and healthy individuals. J. Affect. Disord..

[49] Bouwmans M.E.J., Beltz A.M., Bos E.H., Oldehinkel A.J., de Jonge P., Molenaar P.C.M. (2018). The person-specific interplay of melatonin, affect, and fatigue in the context of sleep and depression. Personal. Individ. Differ..

[50] Thompson R.J., Mata J., Jaeggi S.M., Buschkuehl M., Jonides J., Gotlib I.H. (2012). The everyday emotional experience of adults with major depressive disorder: Examining emotional instability, inertia, and reactivity. J. Abnorm. Psychol..

[51] Putnam K.M., McSweeney L.B. (2008). Depressive symptoms and baseline prefrontal EEG alpha activity: A study utilizing Ecological Momentary Assessment. Biol. Psychol..

[52] Ruscio A.M., Gentes E.L., Jones J.D., Hallion L.S., Coleman E.S., Swendsen J. (2015). Rumination Predicts Heightened Responding to Stressful Life Events in Major Depressive Disorder and Generalized Anxiety Disorder. J. Abnorm. Psychol..

[53] Kircanski K., Thompson R.J., Sorenson J., Sherdell L., Gotlib I.H. (2018). The everyday dynamics of rumination and worry: Precipitant events and affective consequences. Cogn. Emot..

[54] Husky M.M., Mazure C.M., MacIejewski P.K., Swendsen J.D. (2009). Past depression and gender interact to influence emotional reactivity to daily life stress. Cognit. Ther. Res..

[55] Bylsma L.M., Taylor-Clift A., Rottenberg J. (2011). Emotional reactivity to daily events in major and minor depression. J. Abnorm. Psychol..

[56] Kohling J., Moessner M., Ehrenthal J.C., Bauer S., Cierpka M., Kammerer A., Schauenburg H., Dinger U., Köhling J., Moessner M. (2015). Affective Instability and Reactivity in Depressed Patients With and Without Borderline Pathology. J. Personal. Disord..

[57] Slofstra C., Nauta M.H., Holmes E.A., Bos E.H., Wichers M., Batalas N., Klein N.S., Bockting C.L.H. (2018). Exploring the relation between visual mental imagery and affect in the daily life of previously depressed and never depressed individuals. Cogn. Emot..

[58] Hepp J., Lane S.P., Carpenter R.W., Niedtfeld I., Brown W.C., Trull T.J. (2017). Interpersonal Problems and Negative Affect in Borderline Personality and Depressive Disorders in Daily Life. Clin. Psychol. Sci..

[59] Quilty L.C., Watson C., Toneatto T., Bagby R.M. (2017). A Prospective Investigation of Affect, the Desire to Gamble, Gambling Motivations and Gambling Behavior in the Mood Disorders. J. Gambl. Stud..

[60] Fisher A.J., Reeves J.W., Lawyer G., Medaglia J.D., Rubel J.A. (2017). Exploring the idiographic dynamics of mood and anxiety via network analysis. J. Abnorm. Psychol..

[61] Burns M.N., Begale M., Duffecy J., Gergle D., Karr C.J., Giangrande E., Mohr D.C. (2011). Harnessing context sensing to develop a mobile intervention for depression. J. Med. Internet Res..

[62] Kramer I., Simons C.J.P., Hartmann J.A., Menne-Lothmann C., Viechtbauer W., Peeters F., Schruers K., van Bemmel A.L., Myin-Germeys I., Delespaul P. (2014). A therapeutic application of the experience sampling method in the treatment of depression: A randomized controlled trial. World Psychiatry.

[63] Simons C.J.P., Hartmann J.A., Kramer I., Menne-Lothmann C., Höhn P., van Bemmel A.L., Myin-Germeys I., Delespaul P., van Os J., Wichers M. (2015). Effects of momentary self-monitoring on empowerment in a randomized controlled trial in patients with depression. Eur. Psychiatry.

[64] Hartmann J.A., Wichers M., Menne-Lothmann C., Kramer I., Viechtbauer W., Peeters F., Schruers K.R.J., Van Bemmel A.L., Myin-Germeys I., Delespaul P. (2015). Experience sampling-based personalized feedback and Positive affect: A randomized controlled trial in depressed patients. PLoS ONE.

[65] Mohr D.C., Stiles-Shields C., Brenner C., Palac H., Montague E., Kaiser S.M., Carty-Fickes E., Duffecy J. (2015). MedLink: A Mobile Intervention to Address Failure Points in the Treatment of Depression in General Medicine. Int. Conf. Pervasive Comput. Technol. Healthc..

[66] Burton C., Szentagotai Tatar A., McKinstry B., Matheson C., Matu S., Moldovan R., Macnab M., Farrow E., David D., Pagliari C. (2016). Pilot randomised controlled trial of Help4Mood, an embodied virtual agent-based system to support treatment of depression. J. Telemed. Telecare.

[67] Snippe E., Simons C.J.P., Hartmann J.A., Menne-Lothmann C., Kramer I., Booij S.H., Viechtbauer W., Delespaul P., Myin-Germeys I., Wichers M. (2016). Change in daily life behaviors and depression: Within-person and between-person associations. Health Psychol..

[68] Widdershoven R.L.A., Wichers M., Kuppens P., Hartmann J.A., Menne-Lothmann C., Simons C.J.P., Bastiaansen J.A. (2019). Effect of self-monitoring through experience sampling on emotion differentiation in depression. J. Affect. Disord..

[69] Torous J., Staples P., Shanahan M., Lin C., Peck P., Keshavan M., Onnela J.-P. (2015). Utilizing a Personal Smartphone Custom App to Assess the Patient Health Questionnaire-9 (PHQ-9) Depressive Symptoms in Patients With Major Depressive Disorder. JMIR Ment. Health.

[70] Nilsen W.J., Pavel M. (2013). Moving behavioral theories into the 21st century: Technological advancements for improving quality of life. IEEE Pulse.

[71] (2015). Ericsson Consumer Lab Eurupe Ericsson Mobility Report Appendix. Ericsson Mobil. Rep..

[72] Ben-Zeev D., Davis K.E., Kaiser S., Krzsos I., Drake R.E. (2013). Mobile technologies among people with serious mental illness: Opportunities for future services. Adm. Policy Ment. Health.

[73] Colombo D., Cipresso P., Fernández Alvarez J., Garcia Palacios A., Riva G., Botella C. (2018). An Overview of Factors Associated with Adherence and Dropout to Ecological Momentary Assessments in Depression. Annu. Rev. CyberTherapy Telemed..

[74] Karyotaki E., Kleiboer A., Smit F., Turner D.T., Pastor A.M., Andersson G., Berger T., Botella C., Breton J.M., Carlbring P. (2015). Predictors of treatment dropout in self-guided web-based interventions for depression: An “individual patient data” meta-analysis. Psychol. Med..

[75] Kuppens P., Verduyn P. (2017). Emotion dynamics. Curr. Opin. Psychol..

[76] Boswell J.F., Kraus D.R., Miller S.D., Lambert M.J. (2015). Implementing routine outcome monitoring in clinical practice: Benefits, challenges, and solutions. Psychother. Res..

[77] Mikus A., Hoogendoorn M., Rocha A., Gama J., Ruwaard J., Riper H. (2018). Predicting short term mood developments among depressed patients using adherence and ecological momentary assessment data. Internet Interv..

[78] Nuij C., van Ballegooijen W., Ruwaard J., de Beurs D., Mokkenstorm J., van Duijn E., de Winter R.F.P., O’Connor R.C., Smit J.H., Riper H. (2018). Smartphone-based safety planning and self-monitoring for suicidal patients: Rationale and study protocol of the CASPAR (Continuous Assessment for Suicide Prevention And Research) study. Internet Interv..

[79] Saranummi N., Spruijt-Metz D., Intille S.S., Korhonen I., Nilsen W.J., Pavel M. (2013). Moving the science of behavioral change into the 21st century: Part 2. IEEE Pulse.

[80] Brown M., O’Neill N., van Woerden H., Eslambolchilar P., Jones M., John A. (2016). Gamification and Adherence to Web-Based Mental Health Interventions: A Systematic Review. JMIR Ment. Health.

[81] Majumder S., Mondal T., Deen M. (2017). Wearable Sensors for Remote Health Monitoring. Sensors.

[82] Aziz O., Atallah L., Lo B., ElHelw M., Wang L., Yang G.Z., Darzi A. (2007). A pervasive body sensor network for measuring postoperative recovery at home. Surg. Innov..

[83] Patel S., Park H., Bonato P., Chan L., Rodgers M. (2012). A review of wearable sensors and systems with application in rehabilitation. J. Neuroeng. Rehabil..

[84] Ohta S., Nakamoto H., Shinagawa Y., Tanikawa T. (2002). A health monitoring system for elderly people living alone. J. Telemed. Telecare.

[85] Seppälä J., De Vita I., Jämsä T., Miettunen J., Isohanni M., Rubinstein K., Feldman Y., Grasa E., Corripio I., Berdun J. (2019). Mobile Phone and Wearable Sensor-Based mHealth Approaches for Psychiatric Disorders and Symptoms: Systematic Review. JMIR Ment. Health.

[86] Lisetti C.L., Nasoz F. (2004). Using noninvasive wearable computers to recognize human emotions from physiological signals. EURASIP J. Appl. Signal Process..

[87] Choi K.H., Kim J., Kwon O.S., Kim M.J., Ryu Y.H., Park J.E. (2017). Is heart rate variability (HRV) an adequate tool for evaluating human emotions? A focus on the use of the International Affective Picture System (IAPS). Psychiatry Res..

[88] Kocielnik R., Sidorova N., Maggi F.M., Ouwerkerk M., Westerink J.H.D.M. Smart technologies for long-term stress monitoring at work. Proceedings of the CBMS 2013—26th IEEE International Symposium on Computer-Based Medical Systems.

[89] Shen N., Levitan M.-J., Johnson A., Bender J.L., Hamilton-Page M., Jadad A.A.R., Wiljer D. (2015). Finding a Depression App: A Review and Content Analysis of the Depression App Marketplace. JMIR Mhealth Uhealth.

[90] Lin X., Mermelstein R.J., Hedeker D. (2018). A 3-level Bayesian mixed effects location scale model with an application to ecological momentary assessment data. Stat. Med..

[91] Goldfried M.R. (2010). The future of psychotherapy integration: Closing the gap between research and practice. J. Psychother. Integr..

[92] Fernández-Álvarez J., Fernández-Álvarez H., Castonguay L.G. (2018). Summarizing Novel Efforts to Integrate Practice and Research from a Practice Oriented Research Perspective. Rev. Argent. Clín. Psicol..

[93] Walz L.C., Nauta M.H., aan het Rot M. (2014). Experience sampling and ecological momentary assessment for studying the daily lives of patients with anxiety disorders: A systematic review. J. Anxiety Disord..

[94] Schueller S.M., Aguilera A., Mohr D.C. (2017). Ecological momentary interventions for depression and anxiety. Depress. Anxiety.

[95] Yoshiuchi K., Yamamoto Y., Akabayashi A. (2008). Application of ecological momentary assessment in stress-related diseases. Biopsychosoc. Med..

[96] Gee B.L., Griffiths K.M., Gulliver A. (2016). Effectiveness of mobile technologies delivering Ecological Momentary Interventions for stress and anxiety: A systematic review. J. Am. Med. Inform. Assoc..

